# Serum trace element levels and activity of enzymes associated with oxidative stress in endometriosis and endometrial cancer

**DOI:** 10.1002/2211-5463.13738

**Published:** 2023-12-06

**Authors:** Miroslava Rabajdová, Ivana Špaková, Lukáš Smolko, Michaela Abrahamovská, Barbora Baranovičová, Anna Birková, Janka Vašková, Mária Mareková

**Affiliations:** ^1^ Department of Medical and Clinical Biochemistry, Faculty of Medicine P. J. Šafárik University in Košice Slovakia; ^2^ Department of Gynaecology and Obstetrics, Faculty of Medicine P. J. Šafárik University in Košice Slovakia

**Keywords:** copper, gene expression, glutathione, superoxide dismutase, zinc

## Abstract

Endometriosis and endometrial cancer are closely related to oxidative stress. However, the direct relationship between copper and zinc levels and oxidative stress in the extracellular and intracellular space remains unclear. The presented study is focused on the determination of serum Zn and Cu levels, glutathione concentration and enzyme activity in three groups: patients diagnosed with endometrial cancer (EC), patients diagnosed with endometriosis (EM), and a healthy control group. Spectrophotometric determination of trace elements revealed that levels of zinc and copper were lower in blood plasma of patients with endometriosis as compared with the other groups; however, there were no significant differences in the Cu/Zn ratio. Furthermore, significantly increased blood serum glutathione levels were detected in both EM and EC groups compared with the control group. While the activity of superoxide dismutase (SOD) was similar across the studied groups, we observed differences in the activity of other enzymes associated with oxidative stress, including glutathione peroxidase (GPx), glutathione reductase (GR) and glutathione *S*‐transferase (GST), between the control group and the EM and EC patients. Additionally, analysis of gene expression based on free circulating mRNA indicated significant differences in the expression of SOD isoenzymes between the patient groups and the control group; expression of GPx isoenzymes was also altered. Obtained results may have potential application in diagnostics as well as monitoring of endometriosis and endometrial cancer.

## Graphical abstract

Here, we analyzed serum levels of Zn and Cu, glutathione concentration, enzyme activity, and expression of specific genes for antioxidant enzymes based on free circulating mRNA in three groups: (a) patients diagnosed with endometrial cancer, (b) patients diagnosed with endometriosis, and (c) healthy controls.

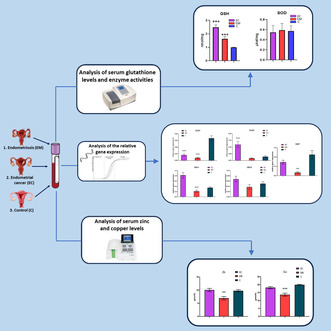

**Abbreviations**
CAT: catalase
EC: endometrial cancer
EM: endometriosis
GPx: glutathione peroxidase
GR: glutathione reductase
GST: glutathione *S*‐transferase
ROS: reactive oxygen species
RT‐PCR: real‐time polymerase chain reaction
SOD: superoxide dismutase

Endometriosis and endometrial cancer of *corpus uteri* belong to the widespread diseases of the female reproductive system closely related to oxidative stress [1]. Reactive oxygen species (ROS) are formed during normal oxygen metabolism and play an important role in cell signaling and homeostasis [2]. Tissue‐specific disruption of the oxidative balance leads to accumulation of free radicals results in chronic inflammatory diseases [3]. Endometriosis is a chronic inflammatory disease characterized by the implantation of endometrial‐like tissue outside of the uterine cavity [4, 5] and belongs to the more prevalent diseases in the reproductive age. Endometrial carcinoma (EC) often develops in the postmenopausal period [6]. Since zinc and copper are integral parts of antioxidant enzymes, their homeostasis is essential for healthy reproductive system [7].

Most of the zinc in human body is stored in skeletal muscle and bones. Only a small amount of it circulates in the bloodstream and binds to proteins [8]. The regulation of intracellular zinc levels is redox sensitive [9]. Zn upregulates the nuclear transcription factor Nrf2, which regulates detoxification [10] and acts as a cofactor of enzymes involved in antioxidant defense as Cu/Zn‐superoxide dismutases [11]. Copper is present in two oxidation states as Cu(I) and Cu(II) and thus exhibits redox activity [12]. Therefore, copper is bound to various proteins in the bloodstream, mainly to ceruloplasmin, albumin, and alpha‐2‐macroglobulin, while the remaining part is found as a cofactor of extracellular superoxide dismutase (SOD3) [13].

According to studies, zinc and copper play a key role in antioxidant and inflammatory processes and also in oncogenesis and malignant transformation of cells [14]. In addition to their individual levels, the Cu/Zn ratio has been studied as a potential diagnostic parameter for inflammatory diseases and cancer [15, 16, 17]. However, the direct relationship between their levels and oxidative stress in extracellular and intracellular space still remains unclear.

## Materials and methods

### Patients and biological material

The patients for this study consisted of two experimental groups and a control group. The experimental groups included 17 women (mean age 34) with histologically confirmed endometriosis (after surgical removal of endometriotic lesions of *corpus uteri*) and 35 women (mean age 60) with histologically confirmed uterine endometrioid carcinoma (31 cases of endometrioid carcinoma and four cases of intramucosal endometrioid carcinoma). The control group included 40 women (average age 39) with negative USG findings of the small pelvis as well as with values of oncomarkers within the reference intervals. Samples from patients with endometriosis of *corpus uteri* were collected at the Department of Gynaecology and Obstetrics of P. J. Šafárik University and UNLP in Košice (Slovakia), and samples from patients with endometrial carcinoma were collected at the Gynaecology Department of the East Slovakian Institute of Oncology as in Košice (Slovakia). The whole blood of the patients was collected on the day of the surgery in BD Vacutainer® sampling tubes, containing anticoagulant additives. The processing of the samples was carried out at the Department of Medical and Clinical Biochemistry of P. J. Šafárik University in Košice. To obtain blood plasma, the blood was centrifuged at 492 ***g***/10 min at 4 °C. For blood serum separation, the blood was collected in a tube without anticoagulant addition and centrifuged at 1507 ***g***/3 min at room temperature. The obtained blood plasma and blood serum were stored at −80 °C prior to the performed analyses. The collection and analysis of the samples were in accordance with the requirements of the Ethics Committee of Faculty of Medicine.

The study was conducted in accordance with the Declaration of Helsinki and approved by the Ethics Committee of Louis Pasteur University Hospital in Košice (2021/EK/10060) and the Faculty of Medicine of P. J. Šafárik University in Košice for studies involving human subjects. Written informed consent was obtained from all subjects involved in the study.

### Analysis of serum zinc and copper levels

The analysis of the serum concentration of Zn and Cu in the studied groups was performed on a clinical chemistry analyzer Randox RX Monza. Commercial colorimetric kits Randox Zinc (ZN2341, detection limit 4.45 μm) and Randox Copper Assay (CU2340, detection limit 6.60 μm) were used according to the recommended manufacturer's instructions. Levels of both trace elements were measured in duplicate for each patient sample. The Randox Acusera human based serum was used to verify the accuracy of the performed analyses with the obtained accuracy of 98.5% for Zn and 98.1% for Cu.

### Analysis of serum glutathione levels and enzyme activities

The activities of antioxidant enzymes superoxide dismutase by SOD determination kit (SOD, EC 1.15.1.1), glutathione peroxidase by Glutathione Peroxidase Cellular Activity Assay kit (GPx, EC 1.19.1.9), glutathione reductase by Glutathione Reductase Assay kit (GR, EC 1.8.1.7), glutathione *S*‐transferase by Glutathione‐*S*‐transferase (GST) Assay kit (EC 2.5.1.18) and levels of reduced glutathione were measured according to the manufacturer's procedures (Sigma‐Aldrich, Taufkirchen, Germany) on an M 501 single beam UV/VIS spectrophotometer (Spectronic Camspec Ltd., Leeds, UK). Results were calculated per protein content determined by bicinchoninic acid assay.

### Analysis of the relative gene expression

Total RNA of analyzed plasma samples was isolated using guanidinium thiocyanate‐phenol‐chloroform extraction, provided as QIAzol Lysis Reagent from Qiagen. Concentration and purity measurements of isolated RNA samples were performed using a Nanodrop LC 2000 (Thermo Scientific, Waltham, USA). A commercial ProtoScript® II First Strand cDNA Synthesis Kit was used to transcribe mRNA into cDNA. Quantification of gene expression was detected by the real‐time PCR method using the SensiMix™ SYBR® No‐ROX kit, using the corresponding specific primer sequences. A Rotor‐Gene Q‐PCR thermocycler (Qiagen, Hilden, Germany) was used for RT‐PCR analysis. Normalization of the results was performed using the β‐actin housekeeping gene. Samples were measured twice for each gene of interest.

### Statistical analysis

The obtained data were analyzed using graphpad prism software version 8 (GraphPad Software, Boston, USA). One‐way ANOVA (nonparametric) Tukey's multiple comparison test was used in the statistical analysis of obtained data. The *P*‐value at the level 0.05 was defined as a statistically significant difference between the examined groups.

## Results

### Serum zinc and copper levels in endometriosis and endometrial cancer

**Fig. 1:**
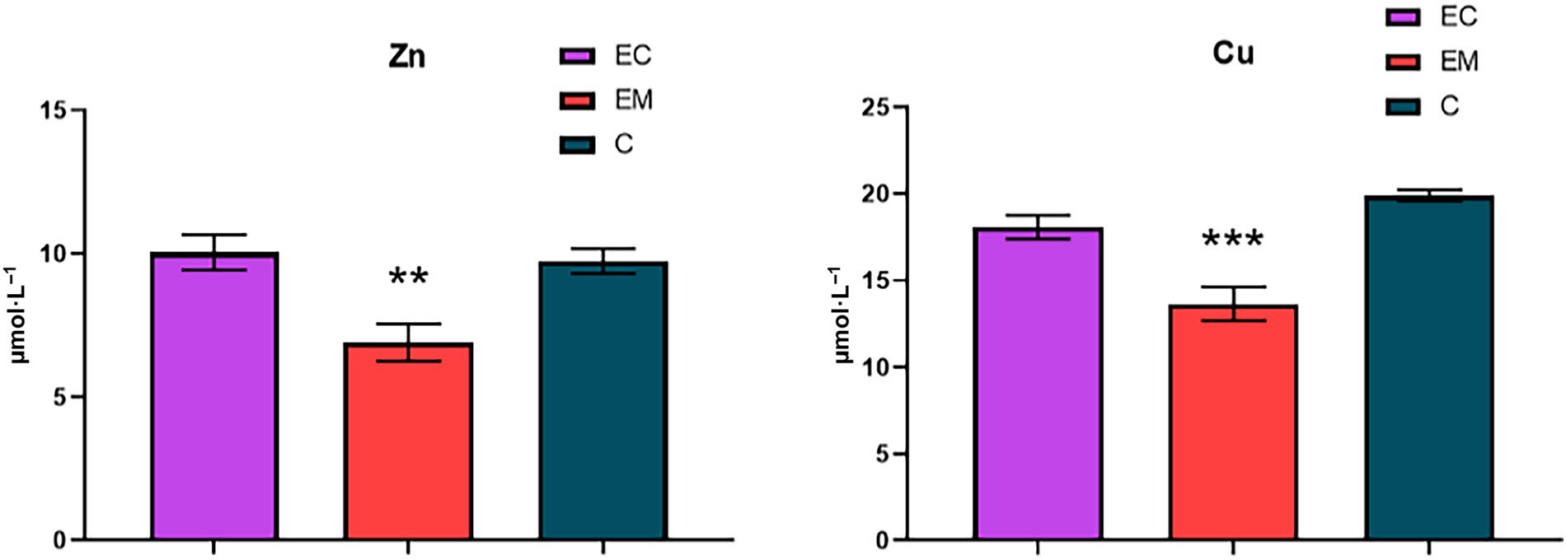
Comparison of serum Zn (left) and Cu (right) levels among the studied groups; EC, endometrial cancer (*n* = 35); EM, endometriosis (*n* = 17); C, control group (*n* = 40). Error bars show SEM. Nonparametric Tukey's multiple comparison test was used for the statistical analysis. Statistically significant differences in comparison with control at ***P < 0.001, **P < 0.01.

Analysis of serum trace element levels in the studied groups was performed by commercially available spectrophotometric kits for zinc and copper, which are designed for routine use in diagnostic laboratories (Fig. 1). A statistically significant decrease in serum Zn (*P* = 0.0068) was observed in samples of women with endometriosis (6.90 ± 0.64 μm); however, no significant difference was found in the samples of patients with endometrial cancer (10.04 ± 0.62 μm) in comparison with the control group (9.74 ± 0.44 μm). A similar trend was observed in the case of Cu levels. Whereas a statistically significant decrease in serum Cu levels (*P* < 0.0001) was found in the samples of patients with endometriosis (13.65 ± 0.97 μm) compared with the healthy controls (19.89 ± 0.31 μm), Cu levels were slightly reduced compared with the healthy controls (18.09 ± 0.69 μm).

**Fig. 2:**
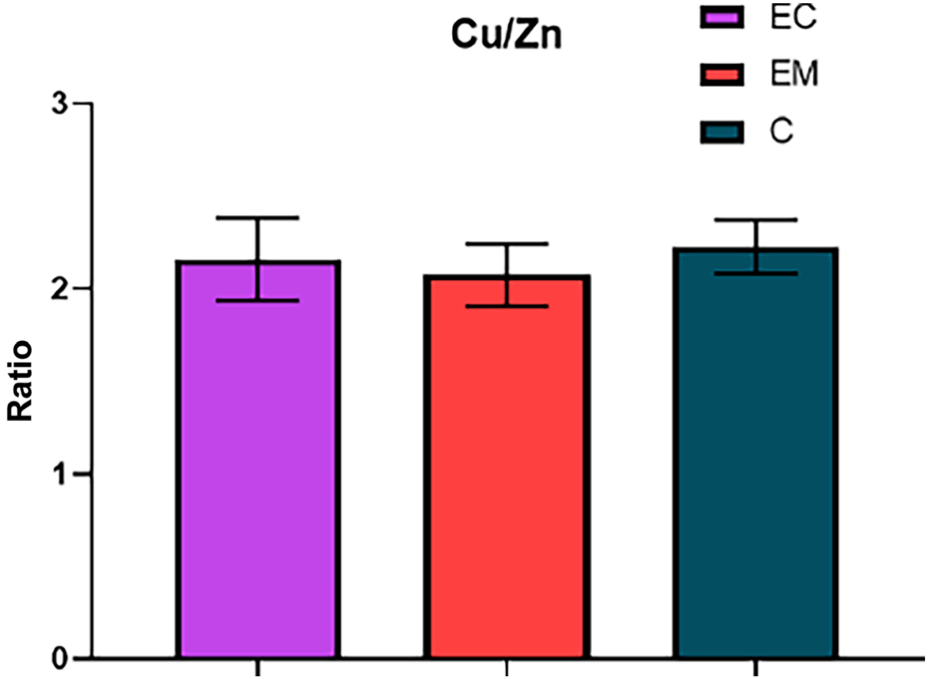
Comparison of the serum Cu/Zn ratio among the studied groups; EC, endometrial cancer (*n* = 35); EM, endometriosis (*n* = 17); C, control group (*n* = 40). Error bars show SEM. Nonparametric Tukey's multiple comparison test was used for the statistical analysis.

Due to simultaneous decrease in both Zn and Cu serum levels in the patients with endometriosis, the Cu/Zn ratio for this group (2.07 ± 0.17) is comparable with the ratio found in patients of endometrial cancer (2.16 ± 0.22) (Fig. 2) and lower than the ratio in the control group (2.22 ± 0.15). The mean values and standard mean errors of the determined elements and their ratio in the studied groups are summarized in Table 1.

**Table 1:** Mean values and standard error of the mean of the trace elements levels in the studied groups.

Group	Zn (μm)	Zn (mg·L^−1^)^a^	Cu (μm)	Cu (mg·L^−1^)^a^	Cu/Zn
Endometrial cancer	10.04 ± 0.62	0.66 ± 0.24	18.09 ± 0.69	1.15 ± 0.26	2.16 ± 0.22
Endometriosis	6.90 ± 0.64	0.45 ± 0.17	13.65 ± 0.97	0.87 ± 0.25	2.07 ± 0.17
Control	9.74 ± 0.44	0.64 ± 0.18	19.89 ± 0.31	1.26 ± 0.16	2.22 ± 0.15

^a^ The values in mg·L^−1^ are shown for better comparison.

### Serum glutathione levels and activity of selected enzymes

To evaluate the actual antioxidant status of the blood serum, the overall activity of SOD along with the concentration of free reduced glutathione and the activities of associated enzymes GPx, GR, and GST were determined (Table 2). The measured values do not show differences in SOD activities between the patients and the healthy controls. In the group of patients with endometrial cancer, there is a visible tendency to decrease GPx activity, and for both groups of patients also for GR, but without significant differences in comparison with the control. The decrease in GST activities compared with the control group is borderline significant, and the trend of decreasing GST activity can also be seen in patients with endometriosis. However, the concentrations of GSH are significantly increased in both groups of patients compared with the control.

**Table 2:** Serum glutathione levels and activity of selected enzymes and standard error of the mean in the studied groups. The values are calculated per mg of total protein content.

Group	GSH (nmol·mg^−1^)	SOD (μkat·mg^−1^)	GPx (μkat·mg^−1^)	GR (μkat·mg^−1^)	GST (μkat·mg^−1^)
Endometrial cancer	2.475 ± 0.199***	0.546 ± 0.133	0.687 ± 0.356	0.321 ± 0.192	0.260 ± 0.117
Endometriosis	1.625 ± 0.171***	0.592 ± 0.131	0.915 ± 0.331	0.320 ± 0.181	0.566 ± 0.227
Control	1.001 ± 0.015	0.575 ± 0.102	0.828 ± 0.299	0.554 ± 0.275	0.874 ± 0.326

Statistically significant differences in comparison with control at ***P < 0.001.

### Gene expression levels based on free circulating mRNA

**Fig. 3:**
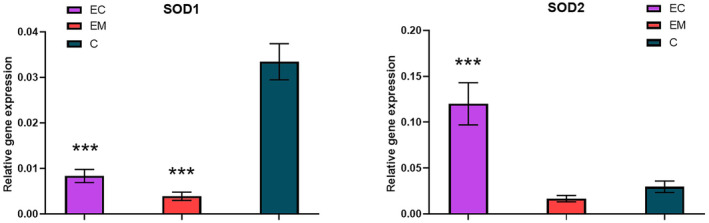
Relative gene expression of SOD1 (left) and SOD2 (right) in the studied groups; EC, endometrial cancer (*n* = 35); EM, endometriosis (*n* = 17); C, control group (*n* = 40). Error bars show SEM. Nonparametric Tukey's multiple comparison test was used for the statistical analysis. Statistically significant differences in comparison with control at ***P < 0.001.

**Fig. 4:**
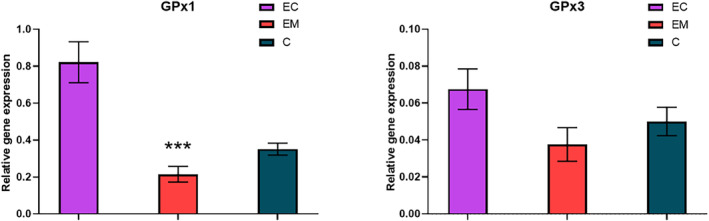
Relative gene expression of GPx1 (left) and GPx3 (right) in the studied groups; EC, endometrial cancer (*n* = 35); EM, endometriosis (*n* = 17); C, control group (*n* = 40). Error bars show SEM. Nonparametric Tukey's multiple comparison test was used for the statistical analysis. Statistically significant differences in comparison with control at ***P < 0.001.

Besides the actual activity of enzymes involved in the antioxidant defense, their gene expression based on the free circulating mRNA was also investigated. A significant decrease in relative gene expression of SOD1 (*P* < 0.0001) was determined in the endometriosis group (0.004 ± 0.001) and in the endometrial cancer group (0.008 ± 0.001) in comparison with the control group (0.033 ± 0.004). However, this trend was not observed in the case of SOD2 relative gene expression (Fig. 3). Although a statistically nonsignificant decrease in SOD2 was found in the samples of the endometriosis group (0.017 ± 0.003) compared with the control group (0.030 ± 0.006), SOD2 levels were significantly increased (*P* = 0.0006) in the samples of the endometrial cancer group (0.120 ± 0.023). The relative gene expression of glutathione peroxidase enzymes GPx1 and GPx3 followed the trend of SOD2 (Fig. 4). The nonsignificant decrease (*P* < 0.0001) in GPx1 relative gene expression was observed in the patients with endometriosis compared with the control group (0.351 ± 0.032) and significant increase in GPx1 relative gene levels (0.215 ± 0.043) in the patients with endometrial cancer (0.822 ± 0.111). The relative gene expression GPx3 was nonsignificantly decreased (0.038 ± 0.009) in patients with and nonsignificantly increased (0.068 ± 0.011) compared with the control group (0.050 ± 0.008).

**Fig. 5:**
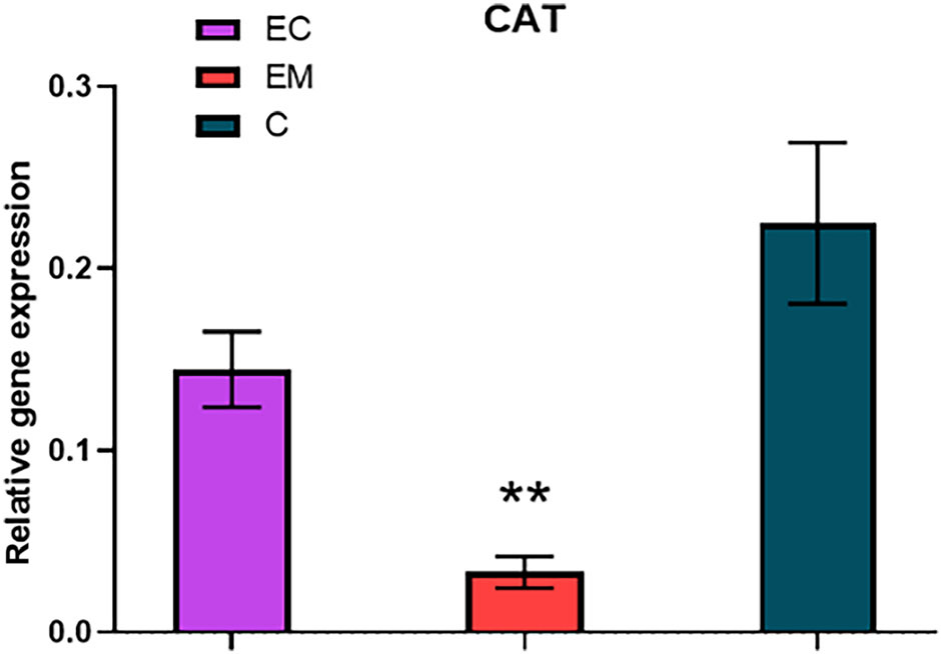
Relative gene expression of CAT in the studied groups; EC, endometrial cancer (*n* = 35); EM, endometriosis (*n* = 17); C, control group (*n* = 40). Error bars show SEM. Nonparametric Tukey's multiple comparison test was used for the statistical analysis. Statistically significant differences in comparison with control at **P < 0.01.

The catalase (CAT) relative gene expression (Fig. 5) showed a tendency of substantial and significant decrease (*P* = 0.003) in the endometriosis group (0.033 ± 0.009) whereas only slightly decreased in the endometrial cancer samples (0.145 ± 0.021) compared with the healthy controls (0.225 ± 0.044).

**Fig. 6:**
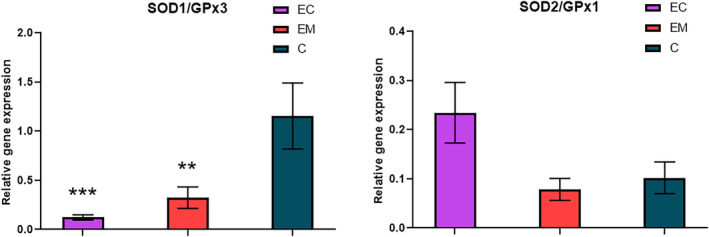
Comparison of SOD1/GPx3 and SOD2/GPx1 ratio in the studied groups; EC, endometrial cancer (*n* = 35); EM, endometriosis (*n* = 17); C, control group (*n* = 40). Error bars show SEM. Nonparametric Tukey's multiple comparison test was used for the statistical analysis. Statistically significant differences in comparison with control at ***P < 0.001, **P < 0.01.

The ratio of SOD/GPx gene expression correlates with monitoring and prognosis of patients with endometrial cancer (Fig. 6). The ratio of SOD1/GPx3 significantly decreased along with the growing prevalence of the endometriosis (*P* = 0.0048; 0.324 ± 0.110) and endometrial cancer (*P* = 0.0003; 0.123 ± 0.027) compared with the healthy controls (1.154 ± 0.336). Accordingly, the ratio of SOD2/GPx1 was found to be nonsignificantly elevated in the group of endometrial cancer (0.234 ± 0.061) compared with the control group (0.102 ± 0.032) and slightly decreased in the patients of endometriosis (0.0079 ± 0.022).

## Discussion

As was mentioned above, Cu and Zn are key essential trace elements for many cellular functions. The levels of these trace elements are under strict homeostatic control mechanisms that regulate their absorption, excretion, and bioavailability. Any imbalance observed in their bioavailability can lead to abnormal cell proliferation and malignant conversion or cell degeneration and apoptosis [18]. In this study, we observed a statistically significant decrease in serum Zn and Cu in samples from women with endometriosis, which correlates with inflammatory conditions related to the higher oxidative stress associated with the disease. Our results also demonstrate slightly lower serum Zn and Cu levels in patients with endometrial cancer compared with the healthy controls. In addition to the Zn and Cu levels, the Cu/Zn ratio is currently considered to be clinically significant since an altered ratio has been reported in various human pathologies associated with inflammation [18, 19, 20]. There are limited data in the literature regarding changes in trace element levels and their relationship to cancers of the female reproductive system. Similar results are shown in the study by Atakul *et al*. [21], who observed lower levels of Zn and Cu as well as comparable Cu/Zn ratio in samples from patients with endometrial cancer compared with healthy controls. On the contrary, another recent and more complex study focused on endometrial diseases reported decreased zinc and increased copper serum levels [22]. Since only a slight decrease in Cu/Zn ratio was found in patients with endometriosis, whereas almost no difference was found between endometrial cancer and control group, the present study does not confirm the significance of this parameter in diagnostics or monitoring of this disease. The differences between the obtained results and other studies may arise from different demographic and genetic factors [23], chemical exposure [24], and lifestyle and diet [25]. Actual serum concentrations of trace elements can also be affected by changes in the levels of metal‐binding proteins in plasma or their increased sequestration in tumor tissues [21]. Another factor that increases the levels of trace elements in the serum is aging since several studies report considerably altered concentrations of Zn and Cu in the group of elderly patients [26]. It is also worth noting that the endometrial cancer group in our study had a significantly higher mean age than the control group due to the increased incidence of endometrial cancer in postmenopausal women [27]. Thus, a slight decrease in Zn and Cu levels observed in the endometrial cancer group in comparison with the control group might not fully reflect the real change.

As Brown *et al*. [28] pointed out, Cu/Zn‐containing SOD1 exists in humans as an apoenzyme that is readily activated by copper without a need for new protein synthesis. Oxidation of SOD1 and the formation of disulfide bonds are essential for its interaction with a metallochaperone and the Cu transfer into its active site. *In vivo* studies have shown that the copper chaperone for SOD1 controls the formation of this disulfide in an oxygen‐responsive step [28]. Although there are still many mechanistic alternatives, it is becoming apparent that the metallochaperone for SOD1, a copper chaperone, does far more than deliver Cu; it has both sulfhydryl oxidase and protein disulfide isomerase activities that appear to allow for higher order types of physiological regulation in response to oxidative stress. The binding of Cu is the rate‐limiting step for the incorporation of Zn with its stabilizing and a positive charge‐providing role [29]. Minimal changes in the overall activity of SOD measured in the serum (SOD1 and SOD3) of the patients with endometriosis and endometrial cancer could be therefore caused by a similar Cu/Zn ratio in these groups as both elements are required for the activation of Cu/Zn SODs. Interestingly, the analysis of SOD1 gene expression based on free circulating mRNA which showed a significant decrease in both the patients with endometrial cancer and endometriosis suggests that the SOD1 have a limited impact on the overall SOD activity in serum. A slight increase in SOD serum activity in the endometriosis group has been also reported by another recent study [30], although older works indicated decreased activity [31]. In contrast, Mn insertion into SOD2 only occurs with newly synthesized and imported molecules into mitochondria [32]. Due to the absence of apoprotein, the higher expression of SOD2 can be therefore considered as a response to the oxidative stress leading to alteration of the metabolic activity of cancer tissue.

An increase in GPx activity along with a decrease in GR activity in the endometriosis group is attributable to the changes of the antioxidant status in the serum. In contrast, both GPx and GR exhibited lower activity in endometrial cancer patients. Arguably, the most significant differences have been found in the serum activity of GST among the studied groups. Decreased activity in comparison with controls was observed in the patients with endometriosis and even more decreased in the patients with endometrial cancer in agreement with the previously published study by Mashayekhi *et al*. [33]. GST activity represents a detoxification mechanism of conjugation of endogenous metabolites as well as potential therapeutic substances with glutathione followed by their subsequent transport out of the cell. Lower GST activities can then reflect chemosensitivity [34] and predict the potential success of further applied therapy. The differences in the activities of enzymes associated with glutathione metabolism in the patients with endometriosis, endometrial cancer, and the control group are also supported by a substantially higher concentration of free reduced glutathione in the serum. Similar results were mentioned by Cecerska‐Heryć *et al*. [35]. Increased GSH levels in cells are associated with cell cycle progression and proliferation. López‐Janiero *et al*. [36] found expression of glutathione synthetase, essential for GSH synthesis, significantly increased in patients with endometrial cancer. Moreover, gamma‐glutamyl transferase (GGT) allowing the efflux of GSH into the cells is expressed on an entire surface of many tumor cells. This provides the tumor cells with an additional source of cysteine and cystine from the breakdown of extracellular glutathione [37]. Increased GSH levels may thus reflect the allowance of survival of cells with altered metabolism [38] and reflect GSH‐associated cytoprotection. Nevertheless, since the free glutathione in the serum is not directly reflecting the redox state inside the endometrial cells, its levels and the activity of the related enzymes (GPx, GR, and GST) in the tissue do not correlate with the results reported herein [39].

The level of gene expression of antioxidant enzymes increases with the Nrf2 transcription factor activity [40] as a response to endogenous/exogenous pro‐oxidant stimuli. The expression of SOD1 is direct to increased median survival of patients with ovarian cancer [41]. In addition, the lower SOD1 expression was found in oocytes of the experimental group with endometriosis [42]. The excessive accumulation of ROS as well as lowered SOD1 expression was determined in endometriotic *cumulus granulosa* cells [43]. In contrast to SOD1, SOD2 expression is upregulated in cancer cells [44] and ovarian cancer cells associated with endometriosis [45]. The level of GPx1/3 is positively regulated with the level of ROS and corresponds to tumorigenicity and tumor stage [36, 46]. The GPx3 expression is also regulated by hypoxic transcription factors [47]. In contrast, an increase in the ratio of SOD2 over GPx1 corresponds to cell proliferation and metastasis [48] whereas the ratio of SOD2/GPx1 is significantly increased in lesion of ectopic endometriosis [49]. In an attempt to investigate the relationship between gene expression and serum trace element levels or enzyme activity, different correlations were performed; however, no significant separation of the respective groups has been revealed (Fig. S1).

With an intention to keep the homogeneity of studied groups of EM and EC, patients with cysts, polycystic ovary syndrome (PCOS), myelomas, ovarian endometriosis, and metastatic cancer were excluded from the investigation. It resulted in a lower number of patients in the respective groups subjected to this study, which might increase the margin of error in the presented data.

## Conclusions

In summary, the presented findings contribute to the complex topic of antioxidative status of serum in the patients affected with the diseases of endometrial tissue of *corpus uteri*. As was mentioned above, a considerable limitation of the study lies in relatively small patient groups and thus only preliminary conclusions can be drawn based on the obtained results. Since the examination of blood represents one of the least invasive and the most convenient diagnostic methods, the presented results including the Zn and Cu levels, the activity of GPx and GST enzymes, and relative gene expressions of selected genes might have potential application in the monitoring of the progression of endometriosis and endometrial cancer. Furthermore, the relationship between the homeostasis of serum trace element levels and the activity of SOD provides an insight into the mechanisms of the antioxidant system. The activities of antioxidant enzymes suggest the need to strengthen the antioxidant defense, which is especially important in the initial stages of disease development. Although this pilot study has revealed some interesting correlations between the trace element levels, activity of antioxidant enzymes, and their gene expression in the blood plasma of patients with gynecological diseases, it is worth noting that the relatively low number of patients subjected to the investigation limits the clinical significance of the results. A full‐scale clinical study would be required for validation of the presented outcomes.

## Conflict of interest

The authors declare no conflict of interest.

## Author contributions

LS, MR, and JV were involved in conceptualization. LS, IŠ, and JV were involved in methodology. MR and MM were involved in validation and supervision. MR, AB, and IŠ were involved in formal analysis. MA and IŠ were involved in investigation. IŠ and BB were involved in resources. MR was involved in data curation and project administration. IŠ, MA, and JV were involved in writing—original draft preparation. LS was involved in writing—review and editing. IŠ was involved in visualization. MM was involved in funding acquisition.

## Supporting information


**Fig. S1.** Correlation of serum Cu levels (above) and Zn levels (below) with SOD1 gene expression for the studied groups. Dashed lines represent correlation trend lines.

## Data Availability

Data are available upon personal request.
