# Supplementary material for: Serum trace element levels and activity of enzymes associated with oxidative stress in endometriosis and endometrial cancer
Source: FEBS Open Bio. 2023 Dec 6;14(1):148–57. doi: 10.1002/2211-5463.13738 (PMC10761925; doi:10.1002/2211-5463.13738)
Supplement: Supplementary file 1 — Fig. S1. Correlation of serum Cu levels (above) and Zn levels (below) with SOD1 gene expression for the studied groups. Dashed lines represent correlation trend lines. [file FEB4-14-148-s001.docx]

Investigation of serum trace element levels and enzymes associated with oxidative stress in selected gynaecological diseases

Miroslava Rabajdová ^1^, Ivana Špaková ^1^, Lukáš Smolko ^1^, Michaela Abrahamovská ^1^, Barbora Baranovičová ^2^, Anna Birková ^1^, Janka Vašková ^1^ and Mária Mareková ^1,^*

^1^ Department of Medical and Clinical Biochemistry, Faculty of Medicine, P. J. Šafárik University in Košice, Trieda SNP 1, 040 11 Košice, Slovakia; miroslava.rabajdova@upjs.sk, 0000-0001-9562-5756; [ivana.spakova@upjs.sk](mailto:ivana.spakova@upjs.sk), [0000-0003-3664-9111](https://orcid.org/0000-0003-3664-9111); [lukas.smolko@upjs.sk](mailto:lukas.smolko@upjs.sk), 0000-0001-9463-6779; [michaela.abrahamovska@student.upjs.sk](mailto:michaela.abrahamovska@student.upjs.sk), 0000-0002-1277-0016; [janka.vaskova@upjs.sk](mailto:janka.vaskova@upjs.sk), 0000-0003-0465-7950; maria.marekova@upjs.sk, 0000-0002-9103-4710

^2^ Department of Gynaecology and Obstetrics, Faculty of Medicine, P. J. Šafárik University in Košice, 040 11 Rastislavova 43, 040 01 Košice, Slovakia; [barbora.kuncova@upjs.sk](mailto:barbora.kuncova@upjs.sk)

***** Correspondence: [maria.marekova@upjs.sk](mailto:maria.marekova@upjs.sk) (M.M.); Tel.: +42-155-234-3368 (M.M.)

**Supplementary Information**

**Fig. S1** Correlation of serum Cu levels (above) and Zn levels (below) with SOD1 gene expression for the studied groups. Dashed lines represent correlation trend lines.
